# An explainable-AI framework reveals novel lncRNAs specific for breast cancer subtypes

**DOI:** 10.3389/fbinf.2026.1760987

**Published:** 2026-03-10

**Authors:** Jai Chand Patel, Avinash Veerappa, Chittibabu Guda

**Affiliations:** 1 Department of Genetics, Cell Biology and Anatomy, University of Nebraska Medical Center, Omaha, NE, United States; 2 Center for Biomedical Informatics, Research and Innovation, University of Nebraska Medical Center, Omaha, NE, United States

**Keywords:** breast cancer subtyping, explainable-AI, long non-coding RNA, multi-omics integration, novel lncRNA

## Abstract

**Background:**

Long non-coding RNAs (lncRNAs) have emerged as important regulators in cancer biology; yet their potential for cancer subtyping remains underexplored particularly in the context of large-scale, multi-class supervised classification frameworks, due to limited publicly available data or their use only as auxiliary features in classification tasks.

**Methods:**

In this study, we utilized an expansive set of 7,177 lncRNAs obtained from 1,021 breast cancer (BRCA) transcriptomics datasets for subtyping using an explainable artificial intelligence (AI) framework. lncRNA, mRNA, and miRNA features were used to build machine learning (ML) models individually and in combination. Four ML classifiers: Naïve Bayes, Random Forest, Artificial Neural Network, and XGBoost were employed to evaluate subtype classification performance.

**Results:**

Using lncRNAs alone, XGBoost demonstrated strong performance with an accuracy of 89.2% and AUROC of 0.99. Addition of miRNA or mRNA features to lncRNA marginally improved the accuracy to 90.8% and 92.2%, respectively, while using all the three features together provided no further gain. A sequential key feature identification pipeline (ANOVA, Boruta, SHAP) has identified interpretable subtype-specific biomarker panels, yielding 119, 66, 54, and 24 unique features for Luminal A, Luminal B, HER2+, and Basal subtypes, respectively. Further lncRNA characterization followed by survival analysis revealed significant subtype-specific novel lncRNAs, including CUFF.25255 (LumA), CUFF.20237 and CUFF.3888 (LumB), CUFF.22414 (HER2+), and CUFF.26607 and CUFF.1961 (Basal).

**Conclusion:**

Our findings highlight the diagnostic and biomarker discovery potential of lncRNAs, and the explainable-AI framework implemented here provides a systematic large-scale evaluation of lncRNA-only and integrative models for multi-class BRCA subtyping for BRCA subtyping and can be adopted to other cancers using the existing cancer transcriptomics data in the public databases.

## 1 Introduction

Long non-coding RNAs (lncRNAs) have emerged as key regulators of gene expression at epigenetic, transcriptional, post-transcriptional and translational levels (Grammatikakis et al., 2014). Mounting evidence shows that they are involved in a wide range of cellular processes including cell differentiation and development. Similarly, dysfunction or aberrant expression of lncRNAs has been associated with hundreds of human ailments including several neurological diseases and cancers (Lin et al., 2024; Tuna et al., 2025). Due to their regulatory roles in various cancer-related processes like cell proliferation, apoptosis, and metastasis, lncRNAs are emerging as promising therapeutic targets for cancer treatment (Coan et al., 2024). Given the tissue-specific nature of transcriptional regulation, lncRNAs could serve as effective biomarkers for a specific cancer or different subtypes of a cancer (Mahato et al., 2024). Motivated by this, our study was designed to test whether lncRNA expression alone can stratify breast cancer subtypes and how the addition of mRNA and miRNA expression data affect the performance of subtype prediction using computational approaches.

Machine learning (ML) techniques have become extremely powerful for refining breast cancer subtype prediction beyond the conventional PAM50 gene panel framework (Ben Rabah et al., 2025; Cancer Genome Atlas, 2012; Cascianelli et al., 2020; Sarkar and Mali, 2022; Wu and Hicks, 2021). Several recent studies have explored deep learning (DL) and graph-based strategies to combine multiple modalities of molecular data. DeepMO, integrating mRNA, DNA methylation, and copy number variations (CNVs), achieved 78.2% accuracy for multi-class subtype classification (Lin et al., 2020). MOGONET, using mRNA, DNA methylation, and miRNA data features, has demonstrated 82.9% accuracy (Wang et al., 2021), while MoGCN, combining mRNA, CNV, and reverse phase protein array (RPPA) data obtained 89.8% accuracy (Li et al., 2022b). On the other hand, moBRCA-net by combining mRNA, DNA methylation, and miRNA data achieved 89.1% accuracy (Choi and Chae, 2023) and Moanna with mRNA, somatic mutations, and CNV data from METABRIC reported 85% accuracy (Lupat et al., 2023). Other hybrid models integrated multiple datatypes such as mRNA, CNV, and histopathology images resulting in 88% accuracy (Liu et al., 2022) and mRNA, CNA, and miRNA resulting in 87.5% accuracy (Cristovao et al., 2022). Recently, we developed GAIN-BRCA framework using mRNA, DNA methylation, and miRNA datatypes, which delivered the highest reported accuracy at 92% (Patel et al., 2025). Notably, none of these high-performing models incorporated lncRNAs, leaving their potential for supervised, multi-class BRCA subtype classification unexplored.

While lncRNAs are increasingly recognized for their regulatory roles in cancer, their potential use in predictive multi-omics models has been notably limited, with very few studies benchmarking their value as standalone transcriptomic features alongside mRNAs, miRNAs and fusion transcripts. Early work using hierarchical clustering revealed subtype-associated lncRNA signatures (Su et al., 2014), while later models incorporate Lasso-Cox, and nomogram frameworks to predict prognosis or identify biomarkers (Li et al., 2022a; Li et al., 2021). Other works proposed pathway- or phenotype-linked lncRNA panels, such as disulfidptosis-associated lncRNAs, where ML models like random forest (RF) and K-nearest neighbor (KNN) have reached AUCs around 0.84–0.87 (Xia et al., 2023); however, only 132 lncRNA features were used in this study. Other approaches, such as LncRIndiv, estimated patient-specific lncRNA expression to capture intra- and inter-subtype variability (Zhao et al., 2021). Additional efforts focused on specific clinical contexts such as diagnostic panels for TNBC (AUC ∼0.80) (Shaath et al., 2021), immune-related lncRNA prognostic models (Liu et al., 2025), and radiomic frameworks linking MRI features with lncRNA signatures (Yu et al., 2023). Moreover, large-scale surveys of subtype- and cell-type-specific lncRNA expression consistently highlight their discriminatory potential across the four subtypes of breast cancer (BRCA), Luminal A, Luminal B, HER2+, and Basal (Bjorklund et al., 2022). Despite this progress, systematic large scale benchmarking of lncRNA-only models for supervised, multi-class subtype classification remains limited, particularly in studies that directly compare lncRNAs against mRNA and miRNA features under a unified machine learning framework. The most prior efforts were predominantly focused on prognostic or binary subtype classification, relied on limited annotations, or incorporated lncRNAs merely as auxiliary features within multi-omics models. Consequently, the independent and integrative discriminatory potential of lncRNAs remains underexplored.

To address these gaps, our study introduces a comprehensive framework that systematically evaluates the predictive and biological value of lncRNAs in breast cancer subtype classification. Rather than claiming novelty based solely on the use of lncRNAs, our contribution lies in the scale and scope of evaluation. We utilized a high-quality in-house curated lncRNA dataset that captures a broader range of biologically relevant lncRNAs across breast cancer subtypes (Guda C, 2025). Using this dataset, we first trained models based solely on lncRNA expression to assess their standalone discriminatory power, followed by integrative modeling with mRNA and miRNA data to examine the additive and synergistic effects. To ensure robust and interpretable results, we implemented a multi-stage key feature identification strategy combining statistical filtering with ANOVA followed by a wrapper-based method, Boruta, and a model-agnostic interpretability tool, SHAP (Zhu et al., 2025; Ahmed et al., 2018; Zhang et al., 2018; Alkuhlani et al., 2017). Overall, our lncRNA-centric study provides a systematic assessment of the independent and combined discriminatory power of lncRNA in classification tasks and reveals several novel lncRNAs for the four BRCA subtypes.

## 2 Materials and methods

**FIGURE 1:**
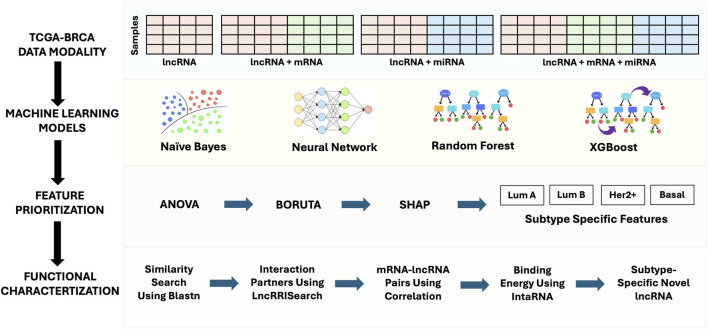
Overview of the analytical framework used in this study. Expression matrices from TCGA-BRCA were prepared in three configurations centered on lncRNA expression: lncRNA alone, lncRNA combined with mRNA, and lncRNA combined with both mRNA and miRNA. These datasets were used to train 4 ML models (Naïve Bayes, Neural Network, Random Forest, and XGBoost) for subtype classification. Feature prioritization was performed sequentially using ANOVA, Boruta, and SHAP to identify subtype-specific molecular markers for Luminal A, Luminal B, HER2+, and Basal subtypes. Functional characterization of selected lncRNAs involved sequence similarity search (BLASTn), interaction analysis using LncRRIsearch, correlation-based mRNA-lncRNA pairing, binding energy estimation with IntaRNA, and annotation of subtype-specific novel lncRNAs.

Patient-matched datasets across three distinct molecular data modalities including lncRNAs, mRNAs, and miRNAs were downloaded for 1,021 tumor and 104 normal samples obtained from the Cancer Genome Atlas (TCGA) BRCA collection. Patient IDs were matched across all data modalities, allowing direct performance comparison across different feature compositions. The pipeline was designed to individually preprocess and normalize each datatype before their use in developing independent and integrative models for ML-based classification. The overall workflow, encompassing data integration, model development, feature prioritization, and functional characterization is summarized in Figure 1.

### 2.1 lncRNA data: extraction and quantification

In this study, we utilized our in-house computational pipeline to extract an expansive high-quality lncRNA dataset (GUDA, 2025), addressing the limited coverage of lncRNAs in standard annotations. BAM files from the TCGA-BRCA dataset were retrieved and converted into paired-end FASTQ files using *samtools* (Li et al., 2009). *STAR* aligner was employed to map reads to the *GRCh38.p14* genome reference using the *gencode.v47.long_noncoding_RNAs.gt*f annotation file (Dobin et al., 2013). The aligned BAM files were processed through *Cufflinks* for transcript assembly (Trapnell et al., 2010). All resulting transcripts were merged into a unified transcriptome file (*merged.gtf*), followed by duplicate removal to obtain *merged_without_duplicates.gtf*. Specifically, transcript entries with redundant coordinates or identical transcript IDs were filtered out. Mitochondrial chromosome annotations were also excluded to improve relevance and reduce noise. Transcript quantification was performed using *featureCounts* from the *subread* package (Liao et al., 2013). Expression profiles were generated for tumor and normal samples separately, yielding count matrices. These raw counts were normalized using *DESeq2’s* variance-stabilizing transformation (VST), log2-transformed, and filtered to retain features with less than 80% missing or zero values (Love et al., 2014). To ensure non-coding purity of the retained transcripts, their coding potential was assessed using the Coding Potential Assessment Tool (CPAT) (Wang et al., 2013). Transcript sequences were extracted from the genome with *gffread*, and CPAT was applied using a species-specific cutoff for humans (0.364). Transcripts exceeding this threshold were removed, and a final high-confidence set of lncRNAs was stored in *filtered_lncRNAs.fa*.

### 2.2 mRNA data processing

mRNA expression data for both tumor and solid normal tissues were downloaded using the *TCGAbiolinks* R package (Colaprico et al., 2016). Raw count matrices were extracted from the GDC portal and merged into a unified expression matrix. A sample metadata table was constructed to assist in tracking and downstream labeling. The data were normalized using *DESeq2’s* variance-stabilizing transformation (VST), log2-transformed, and filtered to remove features with more than 80% missing or zero values (Love et al., 2014). The resulting cleaned mRNA matrix was used for multi-omics modeling.

### 2.3 miRNA data processing

miRNA expression data were processed in the same manner as the mRNA pipeline described above. Count matrices were obtained using *TCGAbiolinks*, merged, and normalized with *DESeq2* VST, followed by log2 transformation and filtering of low-abundance features (>80% zeros or missing) (Love et al., 2014; Colaprico et al., 2016).

### 2.4 Data preparation for ML classification

Preprocessed datasets from the three omics datatypes were used to construct input matrices for ML classification. Each matrix was centered on lncRNA expression: (i) lncRNA alone, (ii) lncRNA combined with mRNA and (iii) lncRNA combined with both mRNA and miRNA. Expression matrices from each omics layer were aligned by patient identifiers and concatenated horizontally to create unified input matrices for downstream modeling, ensuring consistent sample representation across all configurations.

### 2.5 Methodology development

#### 2.5.1 Selection of supervised learning models

We used the four subtypes of TCGA-BRCA patient samples, Luminal A, Luminal B, HER2+, and Basal for building supervised models. Four ML models were selected based on their diversity in learning paradigms, interpretability, and effectiveness in managing high-dimensional transcriptomic contexts. These methods include Naïve bayes (NB), random forest (RF), artificial neural networks (ANNs) and extreme gradient boosting (XGBoost). NB classifiers were implemented using the *GaussianNB* (Webb, 2011) module from scikit-learn (Fabian Pedregosa et al., 2011). Despite the assumption of conditional independence among features, NB often performs well on high-dimensional and sparse datasets, such as gene expression matrices. RF models were constructed using *RandomForestClassifier* with 100 estimators (*n_estimators = 100*) and a fixed seed (*random_state = 42*) (Breiman, 2001). As a tree-based ensemble method, RF can model complex non-linear interactions and offers robustness to overfitting. Another ensemble method, XGBoost classifier, was implemented with the following configuration: *objective = 'multi:softprob’*, *num_class = 4, eval_metric = 'mlogloss’*, and *random_state = 42* (Guestrin, 2016). XGBoost offers efficient and scalable gradient-boosted tree learning and is particularly suited for large-scale structured datasets with redundant or correlated features. ANNs were built using the *TensorFlow/Keras* library (Jain, 1996). The architecture comprised an input layer equal to the number of features, followed by two dense hidden layers with 512 and 256 neurons, respectively, each using *ReLU* activation (Agarap, 2019). Dropout layers with a rate of 0.5 were applied to prevent overfitting. The final output layer consisted of four neurons with *softmax* activation for multi-class prediction (Ian Goodfello, 2016). The network was trained using the Adam optimizer and categorical cross-entropy loss for 50 epochs with a batch size of 32 (Diederik and Kingma, 2014; Anqi Mao and Yutao, 2023). All models were implemented in Python using scikit-learn (Fabian Pedregosa et al., 2011), TensorFlow/Keras, (Martín Abadi et al., 2016; Chollet, 2015), and XGBoost (Guestrin, 2016).

#### 2.5.2 Evaluation metrics

All models were evaluated using stratified 10-fold cross-validation to maintain subtype size balance across folds. Performance metrics, including accuracy, precision, recall, F1-score, and AUROC were computed on each fold and averaged (Powers, 2020; Bradley, 1997; Shakyawar et al., 2022; Mishra et al., 2019; Sethi et al., 2024). ROC curves were generated for each subtype class, and probability estimates from each model were preserved for downstream comparison and interpretability analysis. All performance evaluations were conducted using the full pre-processed feature matrices. Feature selection using the ANOVA-Boruta-SHAP (ABS) pipeline (described below) was not incorporated into model training or cross-validation and was applied only after model benchmarking for feature interpretation and biomarker discovery. This modeling strategy allowed for a robust evaluation of predictive performance of four different ML models across four breast cancer subtypes.

### 2.6 Identification of important subtype-specific key features

Feature identification was performed on the combined lncRNA and mRNA dataset to identify key molecular markers relevant to BRCA subtype classification. Given the high dimensionality of this concatenated dataset (46,715 features), we implemented a sequential feature identification pipeline designed to progressively refine the feature space while reducing noise and redundancy. The ANOVA-Boruta-SHAP, referred to as the ABS pipeline, combined three complementary methods applied in sequence. ANOVA (Analysis of Variance) was used as a univariate statistical filter using the *f_classif* function from the *scikit-learn* library (Lars Sthle, 1989) and features with a *p* < 0.05 were retained. Next, Boruta, a RF-based wrapper method was applied to the ANOVA-filtered features in a one-vs-rest manner for each breast cancer subtype using the *BorutaPy* module in Python (Miron et al., 2010). Finally, SHAP (SHapley Additive exPlanations) was employed to quantify the contribution of each of the Boruta-selected features (Scott Lundberg, 2017). A multiclass XGBoost classifier implemented via the *SHAP* Python library was used to compute SHAP values. Notably, this model was trained solely for SHAP-based interpretation and is distinct from the final predictive classifier used in subtype predictions. This ABS pipeline enabled progressive feature refinement from broad statistical filtering to the identification of key model-based features.

### 2.7 Identification of subtype-enriched pathways

To interpret the functional significance of subtype-specific gene sets, we performed Ingenuity Pathway Analysis (IPA, QIAGEN Inc.). Pathway enrichment was performed using subtype informative mRNA features identified by the ABS pipeline, rather than transcriptomic wide or subtype wise differential expression gene sets. Subtype-specific gene sets identified above were uploaded separately into IPA using default core analysis settings, the Ingenuity Knowledge Base as the reference set; the analysis type set to direct and indirect relationships; and using experimentally observed and high confidence predicted interactions. Since IPA outputs uncorrected p-values for pathway enrichment and does not provide FDR-adjusted results by default, we applied a stringent significance threshold of p < 0.01, corresponding to -log10 (p-value) >2 in IPA outputs, to obtain reliable enriched pathways while minimizing potential false positives.

### 2.8 Survival analysis of subtype-specific mRNAs and lncRNAs

Subtype-specific survival analysis was conducted to evaluate the prognostic relevance of significant protein-coding genes and long non-coding RNAs (lncRNAs) identified from the pipeline described above. For each feature, Kaplan-Meier survival curves were generated (Kaplan and Meier, 1958), and statistical significance was assessed using the log-rank test (Kleinbaum and Klein, 2012; Shakyawar et al., 2024). Rather than employing a fixed median cutoff, we used a more refined data-driven strategy by scanning percentile-based cutoffs between the 20th and 80th percentiles of expression. The optimal threshold was selected based on the minimum log-rank *p*-value, ensuring that both expression-defined groups (high and low) contained at least five patients to maintain statistical power. This approach offered a more precise assessment of the prognostic utility of each candidate biomarker while avoiding arbitrary or biased thresholding.

### 2.9 Novel lncRNA characterization

Subtype-specific lncRNA candidates were first identified using our ANOVA-Boruta-SHAP (ABS) pipeline (Figure 1). We then characterized the putative novel lncRNAs identified for each breast cancer subtype using several approaches described below. Nucleotide sequences of lncRNAs were retrieved in FASTA format from GENCODE v48 and sequence similarity searches were carried out using BLASTN against the human genome (GRCh38.p14.genome.fa) with stringent cutoffs (bit score >40, alignment length ≥200 nt, identity = 100%, e-value <10^−5^) (Altschul et al., 1990). High-identity paralogous RNA sequences identified from this analysis were retained for interaction studies. Next, lncRNA-mRNA interaction analysis was performed using LncRRIsearch (Fukunaga et al., 2019). Here, paralogous RNAs with 100% sequence identity were used as anchors to infer potential mRNA interaction partners (MIPs). The underlying assumption was that if two RNA sequences are identical, they are likely to base-pair with the same mRNA targets when expressed. Based on this, both ABS-derived novel lncRNAs and their paralogous RNAs were used to predict MIPs. Interactions with binding energies ≤ −16 kcal/mol were retained, and the top 100 most stable interactions were selected for each lncRNA. Finally, to assess clinical relevance, we evaluated whether the predicted MIPs of these novel lncRNAs included oncogenes or tumor suppressor genes (TSGs). This was accomplished by cross-referencing with the curated gene catalogue provided by OncoKB database (Chakravarty et al., 2017).

### 2.10 lncRNA-mRNA correlation and base pairing analysis

We computed expression-based correlations between novel lncRNAs and their predicted MIPs using both Pearson and Spearman correlation coefficients (Figure 1). Normalized raw count values for the lncRNAs as well as mRNAs were used to construct expression matrices with patients as rows and either lncRNA (CUFF IDs) or mRNA (gene symbols) as columns. Correlations were then calculated, and pairs with |r| ≥ 0.40 and FDR <0.05 were considered significant. For downstream analysis, we used the mean correlation value of Pearson and Spearman. To validate whether these correlated pairs could physically interact, we extracted the nucleotide sequences of the significant lncRNA-mRNA pairs (CUFF IDs and their targets) from the GENCODE v48 fasta file. Structural feasibility was evaluated using IntaRNA (Mann et al., 2017) which predicts RNA-RNA base-pairing by calculating hybridization events and corresponding binding energy scores (ΔG). Only interactions confirmed by both tools were retained. Stringent parameters such as energy threshold ≤ −16 kcal/mol, a maximum of five interactions per RNA pair, overlap restricted to the lncRNA sequence, no unstacked (lonely) base pairs, at least seven intermolecular base pairs in the seed region, and no GU base pairs or GU ends were applied. Additional folding parameters included: temperature 37 °C (RNAplfold -W), folding window size 150, maximum base-pair distance 100, and Turner-2004 energy model from the ViennaRNA package for base-pair probability estimation. The distribution of binding energies was then plotted to visualize the stability landscape across subtypes (Figure 1). LncRRIsearch, uses IntaRNA output to generate RNA-RNA hybridization images (Fukunaga et al., 2019; Mann et al., 2017). From these distributions, we selected the most stable lncRNA-mRNA interactions for each subtype. Finally, for these top-ranked pairs, LncRRIsearch was used to extract nucleotide-level hybridization images, illustrating specific base-pairing sites and interaction regions.

### 2.11 External cohort transferability analysis using CPTAC cohort

To provide an exploratory assessment of transferability beyond TCGA, we analyzed an independent breast cancer RNA-seq cohort from CPTAC breast cancer cohort of n = 356 (Geffen et al., 2023). Transcripts per million (TPM) values were log-transformed as log2 (TPM+1) for downstream analyses. Because intrinsic subtype labels were not available in CPTAC metadata, PAM50 subtypes were inferred directly from mRNA expression using a centroid-correlation strategy. Briefly, the genefu-based PAM50 centroid matrix was used as the reference. For each sample, Pearson correlation was computed between its log2 (TPM+1) expression profile and each PAM50 centroid; the subtype corresponding to the maximum correlation was assigned as the inferred PAM50 label. Samples classified as Normal-like were excluded from subsequent analyses, yielding a tumor-only cohort comprising of Luminal A (n = 101), Luminal B (n = 129), HER2-enriched (n = 50), and Basal-like tumors (n = 52). Transferability was evaluated by testing subtype-associated expression patterns for a limited subset of gene-level counterparts linked to our prioritized CUFF-level candidates (Table 3): LINC01133 (CUFF.26607/L1RSM), NIFK-AS1 (CUFF.20237/NARUS), and TEC (CUFF.25255/TERCI). For each gene, expression differences across inferred PAM50 subtypes were assessed using the Kruskal–Wallis test on log2 (TPM+1) values, and p-values were adjusted for multiple testing using the BH-FDR across the tested genes. In addition, pairwise subtype comparisons were performed using Wilcoxon rank-sum tests with BH-FDR correction applied within each gene to account for multiple pairwise comparisons.

## 3 Results

Expression matrices were constructed for ML classification using transcriptomic features from 1,021 BRCA patients. After preprocessing and filtering, the final dataset included differentially expressed transcripts from 1,021 BRCA patients, comprising 7,177 lncRNAs, 39,538 mRNAs, and 769 miRNAs. The resulting matrices contained 7,177 features for lncRNA alone, 46,715 features for lncRNA combined with mRNA, and 47,484 features for lncRNA combined with mRNA and miRNA. All datasets maintained identical sample counts and ordering, ensuring that performance differences among models reflected the underlying feature composition rather than sample variability.

### 3.1 lncRNAs exhibit the same level of discriminatory power as mRNAs for subtyping

**FIGURE 2:**
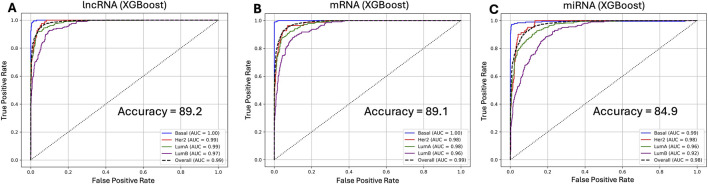
XGBoost ROC curves for single feature set models. **(A)** lncRNA-only, **(B)** mRNA-only, and **(C)** miRNA-only. lncRNA achieve slightly better performance (accuracy = 89.2%, overall AUC = 0.99) with strongest separation for Basal and slightly lower for LumB; miRNA underperforms around an accuracy of 85%.

At first, four different ML models (NB, RF, XGBoost, and ANN) were implemented using lncRNA expression data containing 7,177 features to identify the best performing model for breast cancer subtype classification. Each of these models demonstrated varying degrees of classification performance. NB, a probabilistic model, showed a moderate discriminative ability, achieving an accuracy of 73.9% and an overall AUC of 0.91, yet performing exceptionally well on the Basal subtype (AUC = 0.98) (Table 1; Supplementary Figure S1A). In contrast, the ANN model markedly improved generalization, achieving an accuracy of 87.7% and AUC of 0.98 overall and maintaining consistent performance across the subtypes (AUCs of Basal: 0.99; HER2+: 0.97; Luminal A: 0.97; and Luminal B: 0.95) reflecting its strength in modeling non-linear relationships (Table 1; Supplementary Figure S1B). Random Forest, while also achieving an accuracy of 86.2%, with an overall AUC of 0.98 and a perfect Basal classification (AUC = 1.00) (Table 1; Supplementary Figure S1C). Among all, XGBoost emerged as the best performing classifier, delivering a near-perfect overall AUC of 0.99 and a strong cross-validation accuracy of 89.2% with F1 score of 0.87 (Figure 2A; Table 1). Notably, the Basal subtype was consistently the easiest to distinguish across all models, while Luminal A and B posed greater challenges possibly due to their overlapping transcriptional landscapes. These results highlight the predictive potential of lncRNA expression alone, especially using interpretable and high-capacity classifiers like XGBoost.

**TABLE 1:** Performance of four classifiers across lncRNA, lncRNA combined with miRNA, lncRNA combined with mRNA, and lncRNA combined with mRNA and miRNA feature sets on TCGA-BRCA.

Features used	ML models	Accuracy (%)	Precision	Recall	F1-Score	AUROC
lncRNA (7,177)	Naïve bayes	73.9	0.736	0.796	0.739	0.91
	ANN	87.7	0.875	0.869	0.867	0.98
	Random forest	86.2	0.895	0.752	0.783	0.98
	XGBoost	89.2	0.908	0.850	0.870	0.99
lncRNA + miRNA (7,177 + 769)	Naïve bayes	75.9	0.750	0.80	0.757	0.92
	ANN	88.6	0.867	0.856	0.856	0.98
	Random forest	87.5	0.904	0.774	0.807	0.98
	XGBoost	90.8	0.903	0.858	0.873	0.99
lncRNA + mRNA (7,177 + 39,538)	Naïve bayes	81.0	0.793	0.832	0.801	0.89
	ANN	89.4	0.874	0.858	0.860	0.95
	Random forest	89.3	0.917	0.821	0.849	0.99
	XGBoost	92.2	0.917	0.893	0.902	0.99
lncRNA + mRNA + miRNA (7,177 + 39,538 + 769)	Naïve bayes	81.1	0.800	0.835	0.804	0.89
	ANN	89.9	0.877	0.873	0.872	0.95
	Random forest	89.3	0.915	0.830	0.859	0.99
	XGBoost	92.1	0.920	0.894	0.903	0.99

Values are mean 10-fold cross-validation metrics (Accuracy, Precision, Recall, F1) and multi-class AUROC., lncRNA-only models already perform strongly (XGBoost: Accuracy = 89.2% and AUROC, 0.99). The lncRNA, combined with mRNA, configuration yielded the best overall results (XGBoost: Accuracy = 92.2%, AUROC, 0.99), followed by lncRNA, combined with miRNA (Accuracy = 90.8%, AUROC, 0.99), while including all three data types (lncRNA, mRNA, and miRNA) did not provide further improvement.

Given the superior performance of XGBoost, we selected this model to compare the performance of lncRNA model with those using only mRNA or miRNA features. Using only mRNA features (39,538), the XGBoost model delivered a near identical performance to that built from only-lncRNA features (7,177) with 89.1% accuracy and subtype AUC’s at Basal = 1.00, HER2+/LumA = 0.98, LumB = 0.96) (Figure 2B). In contrast, the miRNA-only model showed weaker performance (accuracy = 84.9%, overall AUC = 0.98) compared to the lncRNA-only model (Figure 2C), which may be attributable to its very small feature size (769). These results demonstrate the remarkable discriminatory potential of lncRNAs as a sole source for breast cancer subtyping.

### 3.2 lncRNA models showed enhanced accuracy in combination with mRNAs or miRNA features

**FIGURE 3:**
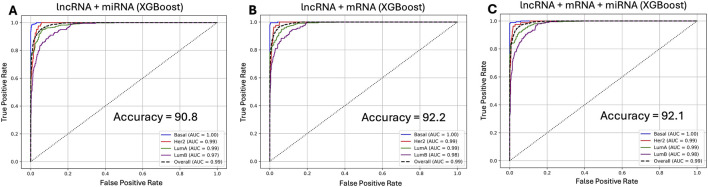
ROC curves showing subtype-level classification performance using the XGBoost model across different data configurations centered on lncRNA expression. **(A)** lncRNA combined with miRNA, **(B)** lncRNA combined with mRNA, and **(C)** lncRNA combined with mRNA and miRNA feature sets. The lncRNA-miRNA model achieved an overall accuracy of 90.8% (AUC = 0.99), the lncRNA–mRNA model reached 92.2% (AUC = 0.99), and the integrative lncRNA-mRNA-miRNA configuration produced comparable performance (92.1% accuracy, AUC = 0.99).

We also tested out pair-wise combinations of the three feature sets with four different ML models (NB, ANN, RF, XGBoost) to identify the best combination of features for breast cancer subtyping. When integrating lncRNA with miRNA and mRNA features separately, a substantial performance boost was observed across all ML models. NB, which previously performed moderately with the single-omics feature sets, has shown markedly improved metrics achieving an average 10-fold cross-validation accuracies of 75.9% and 81% when combined with miRNA and mRNA feature sets, respectively, compared to 73.9% when only-lncRNA data was used (Table 1; Supplementary Figures S2A, S2D). ANN also demonstrated robust predictive capacity with significant improvements in all predictive metrics such as accuracies of 88.6% (lncRNA with miRNA) and 89.4% (lncRNA with mRNA), suggesting enhanced generalization and subtype sensitivity (Table 1; Supplementary Figures S2B, S2E). Similarly, RF benefited from the inclusion of additional features, achieving overall AUROC values of 0.98 (lncRNA with miRNA) and 0.99 (lncRNA with mRNA), outperforming the ANN model (AUROC = 0.95 with mRNA). It reached a perfect AUC for Basal subtype prediction and maintained strong AUC scores for HER2+ (0.99) in both the combinations. The mean accuracies of 87.5% and 89.3% with miRNA and mRNA integration, respectively, compared to 86.2% using lncRNA features alone, confirms its effectiveness for multi-omics learning (Table 1; Supplementary Figures S2C, S2F). Consistent with single omics results, XGBoost continued to outperform all other ML methods achieving an overall AUC of 0.99 and a perfect discrimination for the Basal subtype (AUC = 1.00). It also achieved the highest accuracy of 92.2% for the lncRNA in combination with mRNA features with an F1-score of 0.90. In comparison, the XGBoost model using lncRNA combined with miRNA features delivered only 90.8% accuracy with an F1-score of 0.87 (Figures 3A,B; Table 1). The most notable gain was observed in the Luminal B subtype classification, which had previously lagged in models based on only lncRNA feature set, with an AUC reached to 0.98 with the mRNA feature set. This indicates the complementary nature of mRNA features in improving resolution across challenging subtypes.

### 3.3 Predictive models using the combined features of lncRNA, mRNA, and miRNA

Given the consistently superior performance, XGBoost models were built using features from all three data modalities in a concatenated fashion resulting in a matrix of 1,021 patients and 47,484 features. Surprisingly, the addition of miRNA features to the lncRNA and mRNA framework did not yield any performance gains, suggesting a limited additive value from this third omics layer. All performance metrics as shown in Table 1 across the four models remained nearly identical between the lncRNA plus mRNA and lncRNA, mRNA plus miRNA featured models. For example, with the XGBoost model, the AUROC of 0.99, accuracy of 92.1%, and F1-score of 0.90, virtually mirrored corresponding performance with the lncRNA combined with mRNA model (Table 1; Figure 3C). Similar patterns were observed with the NB, ANN, and RF models (Table 1; Supplementary Figure S3). Collectively, these results underscore the sufficiency of lncRNA and mRNA data as the optimal feature sets for accurate breast cancer subtype classification.

### 3.4 Identification of important subtype-specific features

The best performing XGBoost model utilized a large number of combined features (46,715) that include 7,177 lncRNAs and 39,538 mRNAs from 1,021 patients. However, only a subset of these features is weighted heavily to discriminate the four subtypes. To identify such features, we used a three-step data filtering approach, referred to as ABS pipeline (Section 2.6) using ANOVA, Boruta, and SHAP tools in sequence. Initially, ANOVA identified 41,176 statistically significant features (p-value <0.05), serving as the primary dimensionality reduction filter. These were further filtered first using Boruta and classified using SHAP. Boruta retained 1,336 features for Luminal A, 1,054 for Luminal B, 927 for HER2+, and 828 for Basal. Finally, SHAP analysis distilled them down to 139 for Luminal A, 85 for Luminal B, 60 for HER2+, and 26 for Basal. To improve subtype specificity, we removed features that appeared in more than one subtype class. This filtering step helped isolate features with high discriminatory value, reducing potential ambiguity caused by features commonly associated with multiple subtypes. The final refined sets of unique features are given in Table 2.

**TABLE 2:** Summary of unique protein-coding genes and lncRNAs identified for each breast cancer subtype (LumA, LumB, HER2+, Basal) using the ABS pipeline.

Subtypes	Genes	lncRNAs	Total unique features
LumA	108	11	119
LumB	53	13	66
HER2+	49	5	54
Basal	19	5	24

The totals represent non-overlapping features specific to each subtype, highlighting the molecular distinctiveness of LumA (119 features) versus the compact signature observed in Basal (24 features).

### 3.5 Functional pathway profiling of BRCA subtype-specific mRNA features

Because pathway enrichment was performed on ABS-selected subtype-informative mRNA features rather than on global differentially expressed gene sets, the resulting pathways should be interpreted as molecular programs contributing to subtype discrimination within a supervised learning framework. Consequently, enriched pathways do not necessarily reflect the dominant or canonical biological processes of each breast cancer subtype. Subtype-specific mRNA features presented in Table 2 were used for functional characterization of BRCA subtypes with Ingenuity Pathway Analysis. This analysis was performed to interpret the biological relevance of the mRNA features identified alongside of lncRNA features, providing functional context for the lncRNA-centered classification models. IPA revealed distinct yet overlapping biological themes across breast cancer subtypes, emphasizing pathways critical for cell proliferation, genomic stability, and metabolic regulation. In Luminal A, the most significantly enriched pathways centered around cell cycle regulation, including Cell Cycle Checkpoints (*p-value = 5.8e-07*), Mitotic Metaphase and Anaphase, and p53 signaling, which collectively highlight the involvement of genes such as CDC20, CHEK1, PCNA, and BCL2 in orchestrating DNA replication, damage checkpoints, and apoptotic resistance. Additional pathways included Regulation of TP53 Activity through Phosphorylation and Kinetochore Metaphase Signaling, suggesting coordinated control of chromosomal segregation and tumor suppressor activation within this subtype. These enrichments do not imply a globally high proliferative for Luminal A tumors rather reflect regulated cell cycle and checkpoint components retained within the compact, classification-oriented feature panel that contribute to separating Luminal A from other subtypes. Similarly, for Luminal B, pathway enrichment was dominated by cell cycle and mitotic control pathways, with Mitotic Metaphase and Anaphase (*p-value = 3.1e-06*) and Kinetochore Metaphase Signaling Pathway at the top, implicating genes like BUB1, CCNB1, CENPK, and ESPL1. Furthermore, pathways such as Cell Cycle Checkpoints, Proteasomal PSMD10 Signaling, and Regulation of Apoptosis were significant, suggesting that Luminal B tumors exhibit dysregulation in protein degradation and apoptotic signaling alongside their proliferative drive.

In the Basal subtype, while fewer pathways surpassed the stringent significance threshold, notable enrichment was observed for ESR-mediated signaling (*p-value = 5.1e-05*), involving FOXA1, TFF1, and CXXC5, which is intriguing given Basal tumors’ typical ER-negativity but consistent with reports of partial estrogen pathway activation in subsets. Additionally, UFMylation signaling and ceramide biosynthesis pathways were enriched, driven by genes such as XBP1 and DEGS2, indicating potential alterations in protein homeostasis and lipid metabolism that may underlie aggressive Basal phenotypes. For HER2+ tumors, enriched pathways included Epithelial Membrane Protein Signaling (*p-value = 2.2e-03*), highlighting canonical HER2 (ERBB2) pathway activation alongside IGF1R and ITGA10, as well as metabolic pathways such as the Pentose Phosphate Pathway (Oxidative Branch) and Catecholamine Biosynthesis, driven by G6PD and PNMT. These collectively suggest enhanced proliferative signaling coupled with metabolic reprogramming characteristic of HER2-enriched cancers. Overall, cell cycle and mitotic pathways emerged as the predominant functional themes in Luminal subtypes. In contrast, Basal and HER2+ subtypes demonstrated enrichment in stress adaptation, membrane signaling, and metabolic pathways, underpinned by subtype-specific gene signatures identified in this study. These results provide a mechanistic context for the subtype classification models, supporting their biological validity and offering potential mRNA linked pathways that may interact with or to be regulated by lncRNA signatures.

### 3.6 Prognostic utility of lncRNAs and mRNAs across BRCA subtypes

**FIGURE 4:**
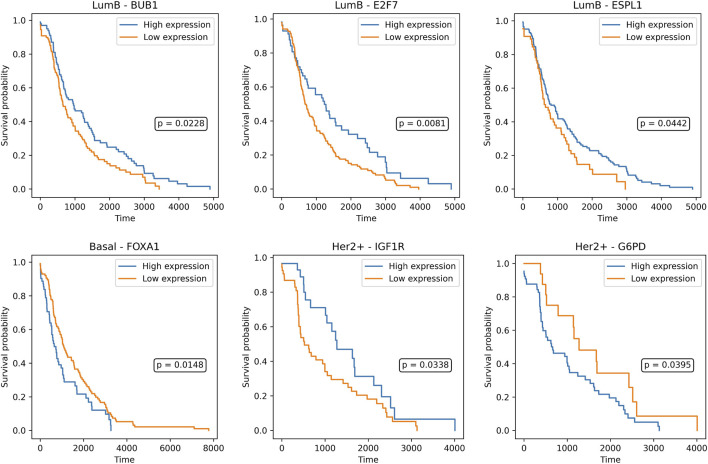
Survival probabilities are shown for high (blue) and low (orange) expression groups, stratified by optimal expression cutoffs. Genes BUB1, E2F7, and ESPL1 demonstrate significant prognostic value in the Luminal B subtype, while FOXA1 is predictive in Basal-like tumors. IGF1R and G6PD show significant survival associations in the HER2+ subtype. Log-rank *p*-values are indicated within each plot.

**FIGURE 5:**
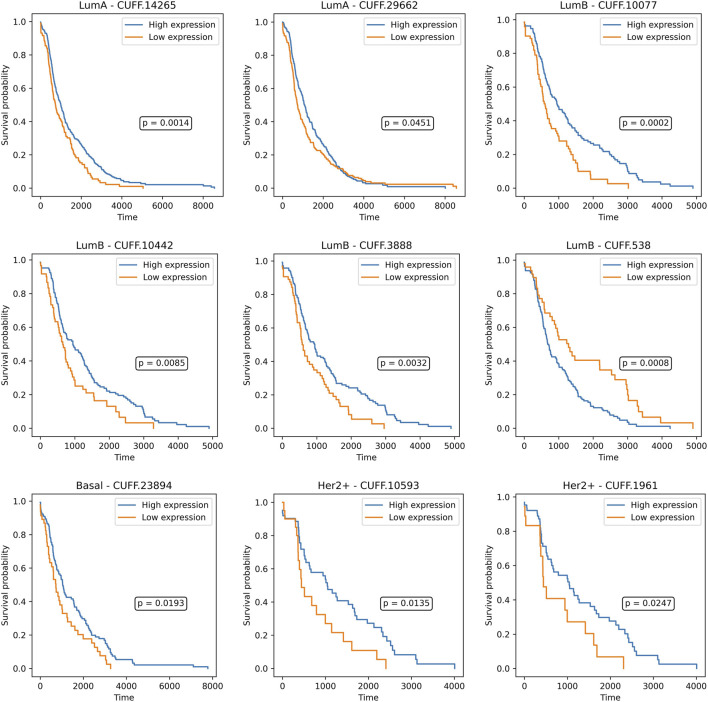
Survival differences between high (blue) and low (orange) expression groups are shown for lncRNAs across Luminal A (CUFF.14265, CUFF.29662), Luminal B (CUFF.10077, CUFF.10442, CUFF.3888, CUFF.538), Basal (CUFF.23894), and HER2+ subtypes (CUFF.10593, CUFF.1961). Each plot presents the survival probability over time, stratified by optimal expression thresholds, with log-rank *p*-values indicating statistical significance.

Survival analysis revealed multiple subtype-specific features with significant prognostic associations, reinforcing their clinical significance. In the Luminal A subtype, while several genes such as *CHEK1, PCNA,* and *CDC20* showed a trend toward prognostic relevance, none reached statistical significance under the log-rank test. Among lncRNAs, *CUFF.14265* (*p* = *0.0014*) and *CUFF.29662* (*p* = *0.0451*) emerged as significantly associated with patient survival, indicating that non-coding elements may contribute more prominently to prognosis in this subtype. In contrast, the Luminal B subtype yielded a richer set of prognostic markers. Genes including *BUB1* (*p* = *0.0228*), *ESPL1* (*p* = *0.0442*), and *E2F7* (*p* = *0.0081*) showed significant survival differences, consistent with their known roles in mitotic regulation. Similarly, lncRNAs such as *CUFF.10077* (*p* = *0.0002*), *CUFF.10442* (*p* = *0.0085*), *CUFF.3888* (*p* = *0.0032*), and *CUFF.538* (*p* = *0.0008*) were significantly associated with outcomes, highlighting a multifaceted regulatory landscape influencing prognosis in Luminal B tumors. In the Basal subtype, *FOXA1* (*p* = 0.0148) emerged as a significant gene-level marker despite the typically reduced number of predictive features in this aggressive subtype. Among lncRNAs, *CUFF.23894* showed a modest but significant association with survival (*p* = 0.0193), suggesting limited but notable non-coding contributions. For the HER2+ group, mRNAs *IGF1R* (*p* = *0.0338*) and *G6PD* (*p* = *0.0395*) showed significant prognostic value, consistent with HER2-driven signaling and metabolic reprogramming. Prognostic lncRNAs in HER2+ included *CUFF.10593* (*p* = *0.0135*), *CUFF.1961* (*p* = *0.0247*), and *CUFF.25223* (*p* = *0.0473*), adding additional layers of subtype-specific risk stratification (Figures 4, 5).

### 3.7 Identification of mRNA targets for novel subtype-specific lncRNAs

From the ABS pipeline, we identified subtype-specific candidate lncRNAs: 124 in LumA, 193 in LumB, 61 in HER2+, and 460 in Basal. BLASTN similarity searches against the human reference genome revealed that a large fraction of these candidates had no prior matches, supporting their classification as putative novel lncRNAs. Paralogous RNA sequences were defined based on the similarity criteria described in the Methods section. Using these criteria, we identified 45 paralogous RNA sequences in LumA, 56 in LumB, 26 in HER2+, and 17 in Basal, which were advanced for interaction analysis. Predicted lncRNA-mRNA interactions showed that LumA (∼2,800 pairs) and LumB (∼3,000 pairs) had the largest networks, while HER2+ (∼1900 pairs) and Basal (∼1,000 pairs) had fewer. Mapping to gene symbols yielded hundreds of unique targets per subtype. Integration with OncoKB identified multiple regulators, including 51 clinically verified genes in LumA, 69 in LumB, 60 in HER2+, and 33 in Basal. Collectively, these findings indicate that novel subtype-specific lncRNAs may regulate key oncogenes and tumor suppressors, providing potential mechanistic insights into breast cancer subtypes.

### 3.8 Refinement of target mRNAs by correlation analysis and structural validation

**FIGURE 6:**
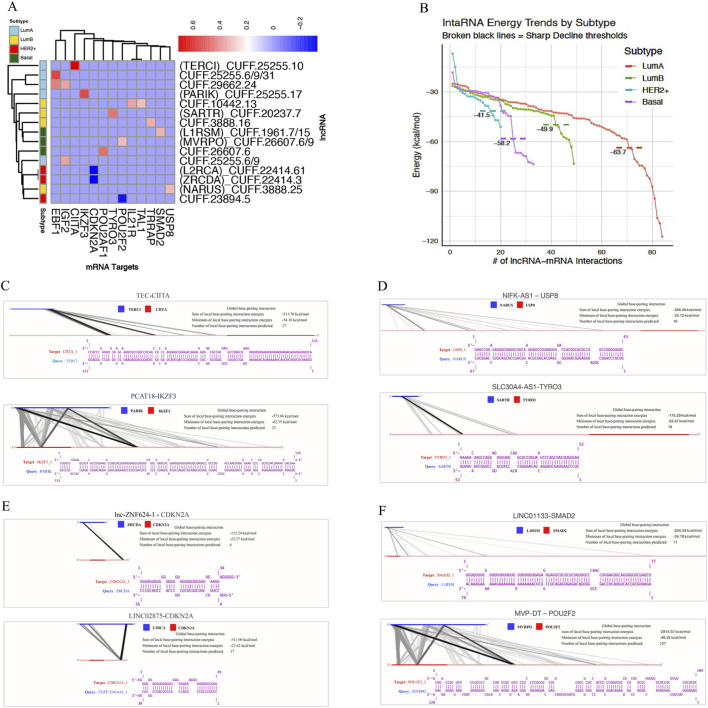
**(A)** Correlation-based heatmap showing the relationships between selected lncRNAs and mRNAs across four molecular subtypes of breast cancer (LumA, LumB, HER2+, and Basal). Color intensity indicates the strength and direction of correlation values between lncRNA–mRNA pairs within each subtype. **(B)** Distribution of intraRNA binding energies across the four subtypes. Broken black lines indicate sharp decline thresholds that distinguish subtype-specific intraRNA energy patterns. **(C–F)** After applying correlation-score and binding-energy filters, the remaining lncRNA–mRNA pairs were visualized to show their hybridization or base-pairing patterns. Each schematic represents the predicted complementary regions between lncRNAs (blue) and their corresponding mRNA targets (red) within the indicated subtype. Distinct interaction topologies highlight potential subtype-specific regulatory mechanisms. The connecting lines represent base-pairing interactions, where darker lines indicate stronger or more stable hybridization.

Correlation analysis revealed numerous subtype-specific lncRNA-mRNA pairs with strong associations. After applying the correlation thresholds (|r| ≥ 0.40, FDR <0.05), we retained nine highly correlated pairs in LumA, five in LumB, and three each in HER2+ and Basal (Figure 6A). To further refine these candidates, we used IntaRNA to generate binding energy distributions and rank interactions by stability. Pairs from LumA and LumB showed steeper declines in their energy plots, indicating a greater proportion of very stable interactions compared to those from HER2+ and Basal. From these distributions, we selected the top-scoring pairs with the most favorable ΔG values (Figure 6B) for each subtype. For these high-confidence pairs, LncRRIsearch was used to generate hybridization maps, which highlighted discrete and stable base-paired regions. These visualizations confirmed that the shortlisted interactions are structurally capable of strong and specific RNA-RNA binding. Notably, eight novel subtype-specific lncRNA-mRNA pairs emerged as the most stable: CUFF.25255-CIITA and CUFF.25255-IKZF3 (LumA); CUFF.20237-USP8 and CUFF.3888-TYRO3 (LumB); CUFF.22414-CDKN2A predicted via BLASTN to be similar to lnc-ZNF624-1 and LINC02875 (HER2+); CUFF.26607-SMAD2 (Basal) and CUFF.1961- POU2F2 (Basal) consistently appeared among the most stable interactions (Figures 6C–F). These interactions involved mRNA targets associated with oncogenes and tumor suppressors, underscoring their potential functional relevance in subtype-specific cancer regulation (Table 3).

**TABLE 3:** Suggested names for novel prognostic lncRNAs.

Subtype	CUFF IDs	Predicted similar gene	Target gene	Suggested new name	Symbol
Luminal A	CUFF.25255	TEC	CIITA	TEC-like -reg-CIITA	TERCI
Luminal A	CUFF.25255	PCAT18	IKZF3	PCAT18-like-reg-IKZF3	PARIK
Luminal B	CUFF.20237	NIFK-AS1	USP8	NIFK-AS1-like-reg-USP8	NARUS
Luminal B	CUFF.3888	SLC30A4-AS1	TYRO3	SLC30A4-AS1-like-reg-TYRO3	SARTR
HER2+	CUFF.22414	Lnc-ZNF624-1	CDKN2A	ZNF624-like-reg-CDKN2A	ZRCDA
HER2+	CUFF.22414	LINC02875	CDKN2A	LINC02875-like-reg-CDKN2A	L2RCA
Basal	CUFF.26607	LINC01133	SMAD2	LINC01133-like-reg-SMAD2	L1RSM
Basal	CUFF.1961	MVP-DT	POU2F2	MVP-DT-like-reg-POU2F2	MVRPO

### 3.9 Proposed nomenclature of novel lncRNAs

Based on similarity and predicted regulatory interactions, we propose new names for novel prognostic lncRNAs identified in our study (Table 1). These names reflect both their reference-like origin and target gene association, providing functional interpretability.

### 3.10 External cohort transferability analysis in CPTAC using inferred PAM50 subtypes

We assessed whether gene-level counterparts linked to prioritized CUFF-level candidates (Table 3) exhibit reproducible subtype-associated expression patterns. Three representative counterparts-LINC01133 (CUFF.26607/L1RSM), NIFK-AS1 (CUFF.20237/NARUS), and TEC (CUFF.25255/TERCI) - showed significant expression differences across the subtypes (Kruskal–Wallis test on log2 (TPM+1), BH-FDR-adjusted p-values: LINC01133 = 3.53 × 10^−7^, NIFK-AS1 = 4.00 × 10^−5^, TEC = 6.53 × 10^−6^; Supplementary Figure S4; Supplementary Table S1). Pairwise Wilcoxon comparisons indicated that the strongest subtype contrasts typically involved Basal-like tumors versus other subtypes, consistent with the marked transcriptional separation of Basal-like disease (Supplementary Table S2). Collectively, these results provide independent-cohort support that a subset of lncRNA-associated signals prioritized in TCGA display reproducible subtype-associated behavior in an external RNA-seq cohort.

### 3.11 Liquid biopsy detectability analysis of candidate lncRNA counterparts

To evaluate whether prioritized lncRNA counterparts are detectable in blood-associated transcriptomic contexts, we queried LINC01133 (CUFF.26607/L1RSM) and TEC (CUFF.25255/TERCI) across publicly available blood-derived expression studies. This meta-level screening identified statistically significant signals for both candidates in peripheral blood and/or bone marrow-associated datasets (Supplementary Table S3; Supplementary Figure S5). Specifically, LINC01133 (CUFF.26607/L1RSM) showed multiple significant blood/bone marrow comparisons (Z ≈ +1.98; p ∼ 6.5 × 10^-4^-3.9 × 10^−3^), while TEC (CUFF.25255/TERCI) exhibited consistent peripheral blood-associated signals with stronger statistical support (Z ≈ +1.09 to +1.21; p ∼ 3 × 10^-6^-7 × 10^-6^). Collectively, these findings indicate that at least a subset of the candidate lncRNA counterparts prioritized by our framework are detectable in blood-associated expression datasets, supporting feasibility for future evaluation in true liquid biopsy analytes and breast cancer-specific clinical settings.

## 4 Discussion

While prior studies have explored lncRNA associated subtype or prognostic signatures in breast cancer, those efforts have largely focused on binary classification tasks, limited lncRNA panels, or outcome prediction, rather than systematic multi-class subtype benchmarking at transcriptome scale. Breast cancer remains a clinically and molecularly heterogeneous disease, with subtype-specific biology influencing prognosis, treatment response, and therapeutic resistance. While traditional gene expression-based classifiers have primarily focused on protein-coding mRNAs, recent discoveries have established long non-coding RNAs (lncRNAs) as central regulators of cancer biology, including proliferation, metastasis, immune evasion, and hormone response (Zhang et al., 2013; Eptaminitaki et al., 2021; Campos-Parra et al., 2018; Mondal and Meeran, 2020; Qi and Du, 2013; Yin et al., 2023; Denaro et al., 2019; Yu et al., 2018; Sikder et al., 2024). Our study supports this growing evidence that lncRNA expression profiles alone can reliably distinguish breast cancer subtypes and serve as effective biomarkers. Notably, lncRNA-based models demonstrated a nearly identical performance to mRNA-only models despite using one-sixth as many features, reinforcing that the non-coding transcriptome contains rich, subtype-specific information that rivals the coding transcriptome. When mRNA features were integrated with lncRNAs, classification performance improved further, the most notable for Luminal B, which is typically difficult to separate due to its mixed expression profile with other subtypes and clinical ambiguity (Ades et al., 2014; Creighton, 2012). The mRNA appears to add complementary resolution to the transcriptomic landscape, helping to refine borderline cases and enhance subtype fidelity. These observations align with previous reports indicating that integrative transcriptomic modeling yields superior predictive and diagnostic accuracy in heterogeneous cancers (Yuan et al., 2019; Wang et al., 2023).

Furthermore, the consistent superiority of XGBoost across all omics configurations highlights the power of gradient boosting in modeling complex transcriptomic relationships. The robustness of these models across 10-fold cross-validation underscores their reliability and generalizability for practical use in clinical settings. Our subtype-specific biomarker discovery framework offers biological interpretability beyond classification. For example, several lncRNAs identified through SHAP-based filtering exhibited strong subtype-enriched expression patterns, warranting follow-up functional studies to investigate their mechanistic roles. These candidates may serve as entry points for unraveling subtype-specific pathways, such as estrogen signaling in Luminal A/B or immune activation in Basal-like tumors (Wasson et al., 2024; Miano et al., 2016; Su et al., 2025; Bjorklund et al., 2022; Mathias et al., 2021). To manage the high dimensionality of transcriptomic data and to prioritize subtype-specific features, we employed a multi-stage ABS pipeline. This layered approach provided a balance of statistical rigor, ML-driven filtering, and model-agnostic interpretability. This pipeline ultimately yielded compact panels of features uniquely informative for each subtype, including a focused set of lncRNAs and mRNAs that hold promise as diagnostic or prognostic biomarkers.

It is important to emphasize that pathway enrichment results in this study arise from supervised, classification-driven feature selection rather than unbiased transcriptome-wide differential expression analyses. Following ABS pipeline, we performed two complementary downstream analyses: (i) functional pathway profiling of mRNA features and (ii) functional characterization of lncRNAs to assess their prognostic and regulatory relevance. IPA analysis on the mRNA components showed clear differences in pathway activity across breast cancer subtypes. Luminal B tumors had strong signals related to cell cycle control and p53 pathways, which fit with their higher growth rates and more frequent p53 changes (Marvalim et al., 2023). Although Luminal A associated feature sets also showed enrichment for cell cycle related pathways, the signals reflect regulated checkpoint and replication associated genes that contribute to subtype discrimination. This is consistent with the known lowerproliferation rates and relatively intact p53 function in Luminal A tumors (Marvalim et al., 2023; Shan et al., 2013). Basal and HER2+ subtypes showed enrichment in stress response, metabolic, and hypoxia-associated signaling pathways, consistent with microenvironmental adaptation (Gatza et al., 2011; Jarman et al., 2019; Kjolle et al., 2023). These subtype-specific enrichments help contextualize the mRNA features within known oncogenic pathways and support biological validity of selected panels.

In parallel, survival analysis and lncRNA characterization were performed on the lncRNA feature sets to evaluate the prognostic and functional significance. Survival analysis used a log-rank based approach that systematically evaluated all possible expression cutoffs, allowing for more sensitive identification of prognostic genes than median-based splits. Both mRNA and lncRNA features yielded subtype-specific markers associated with overall survival. Notably, several lncRNAs from our selected feature sets were found to stratify patients within their respective subtypes, highlighting their translational potential as noncoding prognostic biomarkers. For instance, CUFF.14265 and CUFF.29662 showed prognostic separation in Luminal A, while CUFF.23894 and CUFF.10593 were informative in HER2+ and Basal subtypes, respectively. In Luminal B, both coding genes, such as BUB1, ESPL1, and E2F7, and lncRNAs including CUFF.10077, CUFF.10442, and CUFF.538 were significantly associated with survival, reinforcing the multifactorial nature of prognosis in this aggressive subtype. Similarly, FOXA1 showed prognostic relevance in Basal tumors, and IGF1R and G6PD were informative in HER2+, alongside additional lncRNAs CUFF.1961 and CUFF.25223. For lncRNA characterization, expression correlation with structural interaction prediction, we were able to filter out false positives and ensure that the identified lncRNA-mRNA pairs were both statistically supported and biologically plausible. The integration of OncoKB annotations further strengthened our findings by directly linking several novel lncRNAs to clinically validated oncogenes and tumor suppressors, underscoring their potential translational value. Our proposed naming framework, which connects each lncRNA to both its similarity gene and predicted target, provides a systematic way to interpret and track these biomarkers across studies. Importantly, the averaged scoring strategy based on the mean of Pearson and Spearman correlation coefficients and binding energy values revealed subtype-specific prognostic lncRNAs, with Luminal A and Luminal B subtypes showing particularly stable and confident interactions. Together, these analyses provide a comprehensive regulatory and clinical characterization of lncRNAs derived from ABS pipeline, which could be clinically meaningful lncRNA-mRNA signatures for breast cancer subtypes.

The subtype-specific lncRNA panels identified in this study have potential translational relevance. Compact lncRNA signatures could be incorporated into diagnostic or molecular subtyping assays, either alone or in combination with protein-coding genes, to refine intrinsic subtype classification, particularly in ambiguous cases such as Luminal B tumors. In addition, the association of selected lncRNAs with patient survival within specific subtypes suggests potential utility for risk stratification and prognostic modeling. Finally, the inferred lncRNA–mRNA regulatory relationships provide a framework for generating therapeutic hypotheses, whereby subtype-enriched lncRNAs may modulate key oncogenic pathways and warrant further functional investigation (Zhang et al., 2019).

A limitation of this study is the absence of large-scale external validation of CUFF-level lncRNAs across independent breast cancer cohorts. Widely used resources such as METABRIC are primarily microarray-based and therefore do not provide RNA-seq data that can be re-processed using the same transcriptome assembly and quantification strategy applied in this study. As a result, many CUFF-level lncRNAs identified here are not represented on array platforms, limiting the feasibility of direct external validation using such datasets.

## 5 Conclusion

This study underscores the emerging significance of long non-coding RNAs (lncRNAs) as robust transcriptomic markers for breast cancer subtyping. By leveraging a robust multi-omics dataset and evaluating multiple ML models, we demonstrate that lncRNA expression alone achieves high predictive performance, particularly when modeled using XGBoost, which consistently outperformed other classifiers. Integrating lncRNA with mRNA features further enhanced classification and interpretability, most notably for the clinically challenging Luminal B subtype. The ABS framework enabled the identification of potential lncRNA-centered subtype-specific biomarker panels that retained high discriminative power while offering biological interpretability. Overall, these findings position the lncRNAs as functional regulators and promising biomarkers that merit further experimental validation for their potential role in precision oncology.

## Data Availability

Publicly available datasets were analyzed in this study. This data can be found here: https://portal.gdc.cancer.gov/; https://zenodo.org/records/17820359.
