# Supplementary material for: An explainable-AI framework reveals novel lncRNAs specific for breast cancer subtypes
Source: Front Bioinform. 2026 Mar 10;6:1760987. doi: 10.3389/fbinf.2026.1760987 (PMC13008977; doi:10.3389/fbinf.2026.1760987)
Supplement: Supplementary file 1 [file Supplementaryfile1.docx]

Supplementary Material

1. **Supplementary Figures**


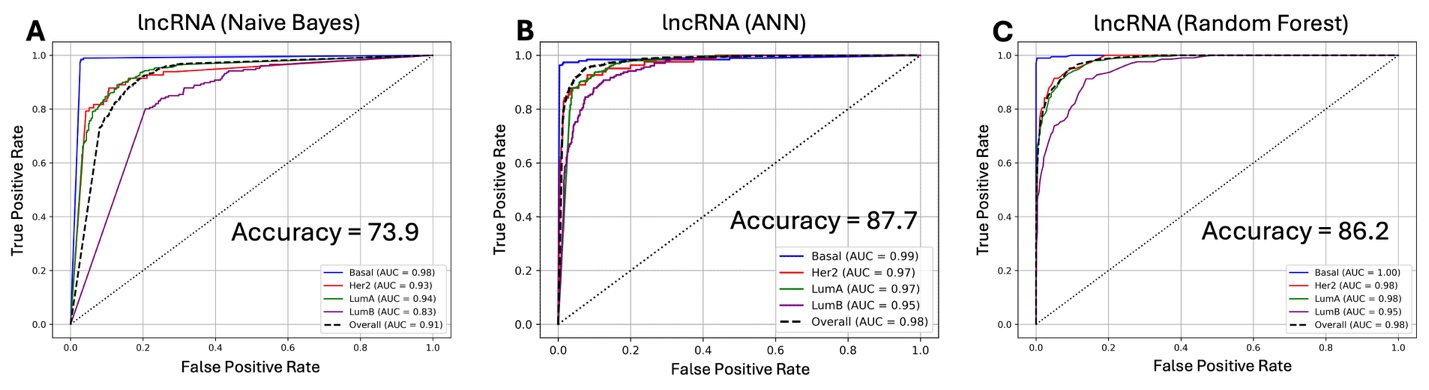


**Supplementary Figure 1.** Receiver operating characteristic (ROC) curves showing the classification performance of lncRNA-based models using Naive Bayes, Artificial Neural Network (ANN), and Random Forest algorithms. The Naive Bayes model achieved an accuracy of 73.9%, while the ANN and Random Forest models reached 87.7% and 86.2% accuracy, respectively, indicating that ANN and Random Forest provided better predictive performance compared to Naive Bayes.


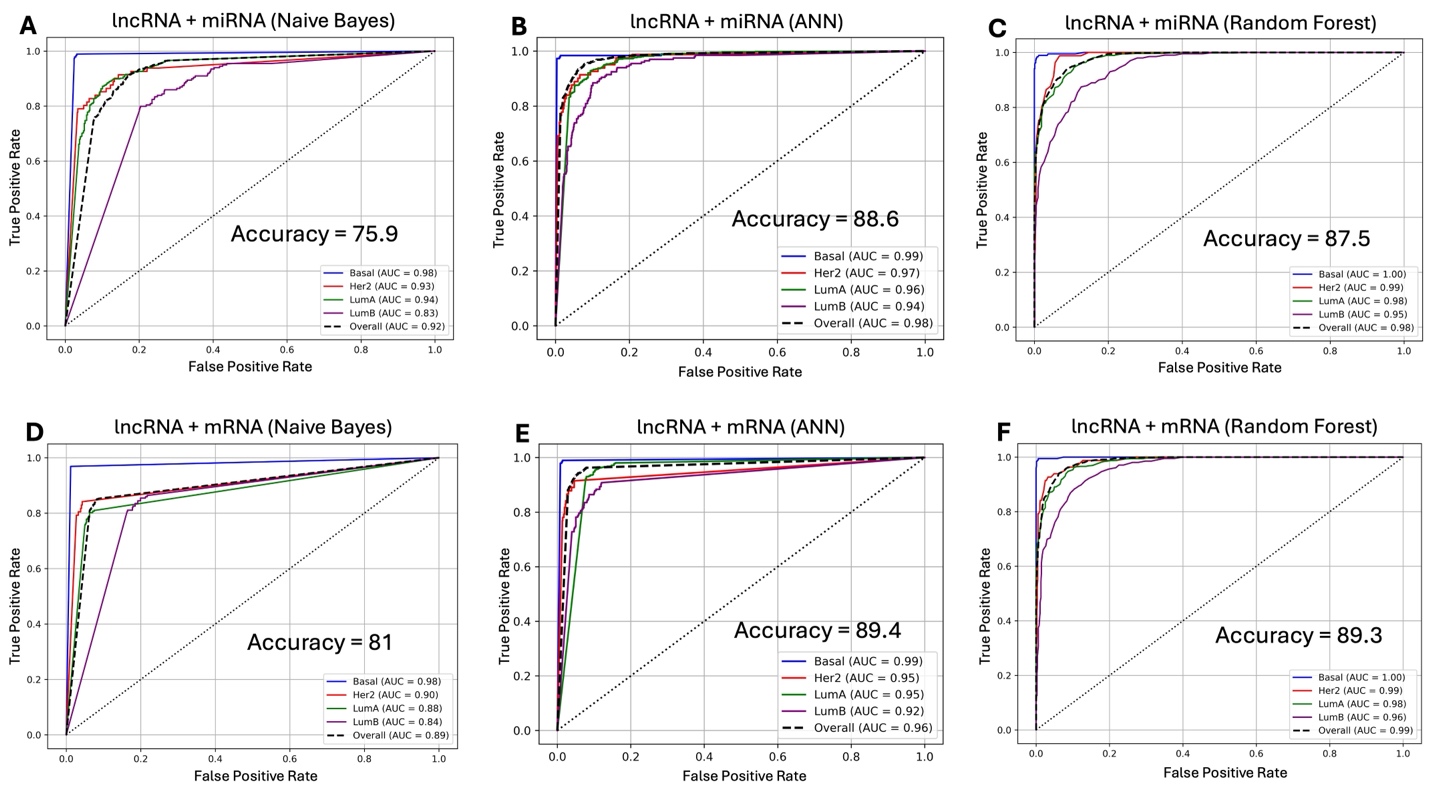


**Supplementary Figure 2.** Receiver operating characteristic (ROC) curves showing the performance of combined lncRNA, miRNA, and mRNA models across three machine learning algorithms. Panels A–C represent models using lncRNA + miRNA features with Naive Bayes (accuracy = 75.9%), Artificial Neural Network (ANN; accuracy = 88.6%), and Random Forest (accuracy = 87.5%), respectively. Panels D–F show models integrating lncRNA + mRNA features with Naive Bayes (accuracy = 81%), ANN (accuracy = 85.4%), and Random Forest (accuracy = 89.3%). Overall, ANN and Random Forest models demonstrate higher predictive accuracy compared to Naive Bayes for both feature combinations.


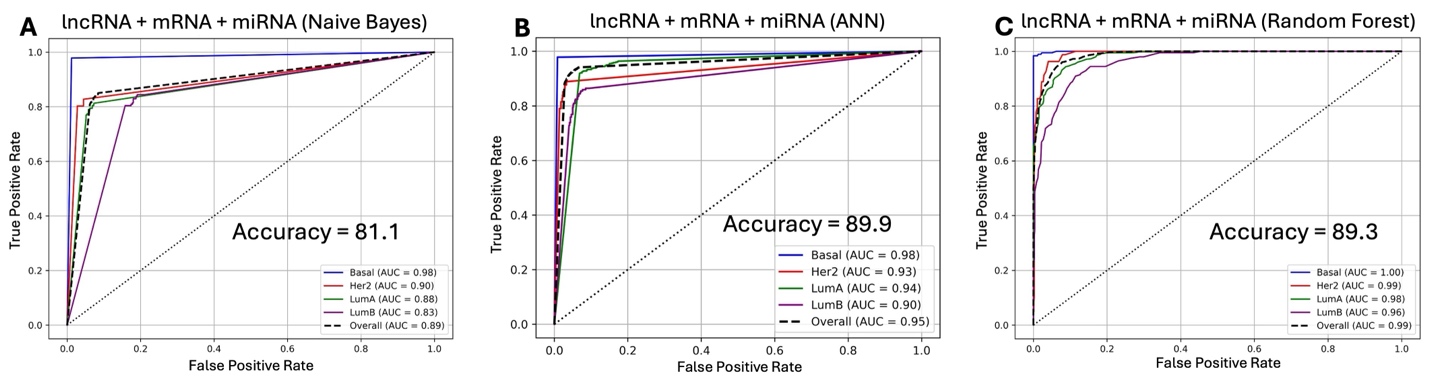


**Supplementary Figure 3.** Receiver operating characteristic (ROC) curves showing the classification performance of models combining lncRNA, miRNA, and mRNA features using three machine learning algorithms. (A) Naive Bayes model achieved an accuracy of 81.1%, (B) Artificial Neural Network (ANN) model reached 89.9%, and (C) Random Forest model achieved 89.3%. The results indicate that integrating all three RNA types improves model performance, with ANN and Random Forest showing the highest predictive accuracy.
